# Polypharmacy and potentially inappropriate medications among elderly patients in the geriatric department at a single-center in China: A retrospective cross-sectional study

**DOI:** 10.1097/MD.0000000000027494

**Published:** 2021-10-22

**Authors:** Lina Tao, Xiaoyu Qu, Huan Gao, Jinghui Zhai, Yueming Zhang, Yanqing Song

**Affiliations:** Department of Pharmacy, The First Hospital of Jilin University, Changchun, China.

**Keywords:** Beers criteria, elderly, hyperpolypharmacy, polypharmacy, potentially inappropriate medication

## Abstract

The aging of the population has become a worldwide concern, especially in China. Polypharmacy and potentially inappropriate medications (PIMs) are prominent issues in elderly patients. Therefore, the aim of this study was to investigate the prevalence of polypharmacy and PIMs in older inpatients and further to explore the factors associated with PIM use.

A retrospective, single-center, cross-sectional study was conducted. A total of 1200 inpatients aged 65 years or older admitted from January 2015 to December 2015 were included. The prevalence of polypharmacy (5–9 medications) and hyperpolypharmacy (10 or more medications) was calculated. The 2019 American Geriatric Society Beers criteria were applied to assess PIMs use. Multivariate logistic regression was used to determine the independent factors of PIM use, while zero-inflated negative binomial regression was performed to evaluate the relationship between polypharmacy and PIM use.

The median age of the study population was 76 years (interquartile range = 71–81). The median number of medications was 9 (interquartile range = 7–12). 91.58% of the patients took 5 or more medications simultaneously, and 30.08% of the patients were subjected to one or more PIMs. Spironolactone, furosemide, and zopiclone were the top 3 most frequently encountered PIMs. Hyperpolypharmacy and older age were identified as independent factors associated with PIM use. The risk of PIMs rises with the number of medications prescribed.

Polypharmacy and PIM use were common in our study, and the risk of PIM use correlated with an increase in the number of medications already prescribed.

## 1 Introduction

Population aging is becoming a common problem worldwide, and especially in China. In 2015, the proportion of the Chinese population aged 60 or older reached 10.5%, with an aging society being defined as more than 7% of the population over 60.^[1,2]^ Moreover, the number of elderly individuals is predicted to reach 483 million by 2050, accounting for 34.1% of the total population of China.^[3]^

With aging, the majority of elderly individuals have multiple comorbidities. Consequently, many older people receive a combination of medications, resulting in a practice called polypharmacy.^[4]^ Polypharmacy, defined as “the use of multiple drugs or more than are medically necessary,” is common in older adults.^[5,6]^ Polypharmacy is associated with an increased risk of adverse drug reactions, and adverse drug–drug and drug–disease interactions.^[7,8]^

Potentially inappropriate medications (PIMs) are defined as medications whose adverse risks exceed their health benefits when compared with alternative therapies.^[9,10]^ Several tools have been developed to identify PIM use in the elderly, and the Beers criteria (initially published in 1991^[11]^ and then updated in 1997, 2003, 2012, 2015, and 2019^[12–16]^), being one of the most popular guidelines for evaluating PIM use in older people.

A large number of studies have shown that PIM use in the elderly was associated with detrimental health outcomes such as adverse drug reactions, hospitalization, and death.^[17–20]^ Furthermore, PIM use can result in higher levels of health service utilization and raise associated care costs.^[21,22]^ Therefore, it is important to monitor and minimize the prevalence of PIM use among the elderly.

Numerous studies conducted worldwide have evaluated the prevalence of PIM use in the elderly. In the United States and other developed countries, the prevalence of PIM use ranged from 14% to 43%.^[23]^

PIM use in elderly patients has attracted public attention recently in China.^[24,25]^ Hence, the aim of this study was to investigate the prevalence of polypharmacy and PIM use and the factors associated with PIM use in elderly inpatients aged 65 or older, who were admitted to the geriatric department of the First Hospital of Jilin University using the 2019 Beers criteria.

## 2 Methods

### 2.1 Study design and ethical considerations

This was a retrospective, single-center, cross-sectional study, and was reported in accord with the Strengthening Reporting of Observational Studies in Epidemiology statement.^[26]^ The study was conducted in the geriatric department of the First Hospital of Jilin University, Changchun, China, a tertiary care teaching hospital. The geriatric department treats patients with age-associated chronic conditions, such as cardiovascular, cerebrovascular, and respiratory diseases. The study was approved by the Medical Ethical Committee of the First Hospital of Jilin University and was conducted according to the Declaration of Helsinki. Given that the study used data collected from electronic medical records (EMRs) and the fact that the patients were not contacted, gathering individual informed consent was not a requirement imposed by the Medical Ethical Committee of the First Hospital of Jilin University.

### 2.2 Setting and participants

This retrospective, cross-sectional study utilized EMRs of patients admitted to the geriatric department in the First Hospital of Jilin University between January 1st and December 31th, 2015. 1200 consecutive patients aged 65 or older who received at least 1 medication during hospitalization were included in the study. Patients were excluded if they were not receiving any medication during the data collection period.^[27]^

### 2.3 Data collection

The EMRs of the patients were retrieved from the hospital information management system of the First Hospital of Jilin University. The retrieved information included gender, age, diagnosis, a list of prescribed medications, and corresponding dosages. Since, generally, self-medication is not permitted in the hospital, details of all medications prescribed during hospitalization are included in the EMRs, comprising oral medications and injections. However, topical medications, inhaled drugs, and eye drops were excluded. Medications for the treatment of chronic and transient diseases were included. Medications were coded using the Anatomical Therapeutic Chemical Classification system.^[28]^ Disease diagnoses were derived from the diagnostic information or disease condition comprehensively recorded in the EMRs. Data extracted from the EMRs were recorded on a spreadsheet using Microsoft Excel software. Two authors extracted and analyzed the relevant data independently. Disagreements were resolved by discussion with a third author.

### 2.4 Outcome measures

The primary outcome measure was the prevalence of polypharmacy and PIM use. Polypharmacy refers to the application of multiple medications, but there is no universal standard definition. The most commonly reported definition of polypharmacy (and the definition of polypharmacy chosen for this study) alludes to an individual being prescribed 5 or more medications.^[29]^ In our study, hyperpolypharmacy is subsequently defined as taking 10 or more medications.^[27]^ Prescriptions were categorized into 3 tiers according to the number of prescribed medications: 1 to 4 medications (no polypharmacy), 5 to 9 medications (polypharmacy), and 10 or more medications (hyperpolypharmacy).^[6]^ PIM use was defined based on the 2019 Beers criteria of American Geriatric Society.^[15]^ All 5 components of the 2019 Beers criteria were utilized to screen for PIM use:

i) PIMs in older adults,
ii) drug-disease and drug-syndrome PIMs,
iii) PIMs to be used with caution,
iv) drug–drug interactions, and
v) PIMs based on kidney function.

The PIMs was evaluated for the entire duration of hospitalization among all the participants who were eligible for this study. The list of PIMs detected among patients was compiled and their prevalence was calculated.

### 2.5 Statistical analysis

Continuous data were described in terms of mean (standard deviation) or the median (interquartile range, IQR) according to normal or skewed distribution. Categorical data were described as numbers and proportions. The chi-square test was applied to compare nominal categorical variables and the Mann–Whitney *U* test was used to compare ordinal categorical variables. Student's *t* test was applied to compare normally distributed continuous variables and the Mann–Whitney *U* test was used to compare skewed continuous variables. To identify independent factors associated with PIM use, multiple logistic regression analysis was conducted. Variables such as gender, age, length of hospital stay, the number of diagnosed diseases, and the number of prescribed medications were included in the multiple logistic regression model. Zero-inflated model was used to account for frequent zero-value observations and overdispersed data. Zero-inflated negative binomial regression was used to calculate the incidence rate ratio for PIM use across polypharmacy. To evaluate the nonlinear associations between the risk of PIM use and the number of medications prescribed, the total number of medications was modeled as a cubic spline using 5 medications as a reference, adjusted for age, gender, length of hospital stay, and the number of diagnosed diseases. The minimum sample size was estimated to be 1191 patients for a two-sided 95% confidence interval with a width equal to 0.050 when the sample proportion of PIMs was 25%, which was deemed to be sufficient to detect PIMs among older adults based on previous published studies. Statistical analyses were performed using SPSS version 22.0 (IBM, SPSS Inc.) and Stata 15 (StataCorp LLC, 2015; College Station, TX) software. All *P* values shown were two-sided and statistical significance was defined as *P* < .05.

## 3 Results

### 3.1 Characteristics of the study population

During the study period, 1216 patients aged 65 years or older were admitted. Of these, 16 patients were excluded because of no medication was prescribed during hospitalization. A total of 1200 patients were thus enrolled in this study. The median age of the participants was 76 years (IQR = 71–81), of which 57.58% were female. The median length of hospital stay was 8 days (IQR = 6–11) (Table 1). Of all patients, 20 ADRs occurred, and 18 ADRs were due to PIMs. Hyponatremia caused by diuretics and selective serotonin reuptake inhibitors were the 18 ADRs.

**Table 1:** Characteristics of 1200 elderly participants identified based on the 2019 Beers criteria.

Variables	Overall (n = 1 200)	PIM (n = 361)	Non-PIM (n = 839)	*P* value
Gender (n [%])				.911^∗^
Female	691 (57.58)	207 (57.34)	484 (57.69)	
Male	509 (42.42)	154 (42.66)	355 (42.31)	
Age (yrs) (n [%])				<.001^∗^
65–74	507 (42.25)	109 (30.19)	398 (47.44)	
75–84	543 (45.25)	188 (52.08)	355 (42.31)	
≥85	150 (12.50)	64 (17.73)	86 (10.25)	
Age (yrs) (median [IQR])	76 (71–81)	78 (73–82)	75 (70–80)	<.001^†^
Length of hospital stay (d) (n [%])				.010^∗^
1–5	234 (19.50)	52 (14.40)	182 (21.69)	
6–10	629 (52.42)	196 (54.30)	433 (51.61)	
≥11	337 (28.08)	113 (31.30)	224 (26.70)	
Length of hospital stay (d) (median [IQR])	8 (6–11)	9 (7–11)	8 (6–11)	.001^†^
No. diagnosed disease (n [%])				<.001^∗^
1–5	393 (32.75)	82 (22.71)	311 (37.07)	
6–10	645 (53.75)	214 (59.28)	431 (51.37)	
≥11	162 (13.50)	65 (18.01)	97 (11.56)	
No. diagnosed disease (median [IQR])	7 (5–9)	8 (6–10)	6 (5–9)	<.001^†^
No. prescribed medication (n [%])				<.001^∗^
1–4	101 (8.42)	8 (2.22)	93 (11.08)	
5–9	529 (44.08)	76 (21.05)	453 (53.99)	
≥10	570 (47.50)	277 (76.73)	293 (34.92)	
No. prescribed medication (median [IQR])	9 (7–12)	12 (10–15)	8 (6–11)	<.001^†^

### 3.2 Comorbidity

Participants typically had 7 comorbidities (IQR = 5–9). Overall, 32.75% (n = 393), 53.75% (n = 645), and 13.50% (n = 162) of the participants had 1 to 5, 6 to 10, or 11 or more comorbidities, respectively (Table 1). The majority of the participants had hypertension (56.67%) and 56.42%, 50.08%, and 32.92% had coronary vascular disease, cerebrovascular disease, and infectious disease, respectively (Table 2).

**Table 2:** The prevalence of various diseases diagnosed within the study population.

Diagnosis	Patients (n [%])
Hypertension	680 (56.67)
Coronary vascular disease	677 (56.42)
Cerebrovascular disease	601 (50.08)
Infectious disease	395 (32.92)
Diabetes	287 (23.92)
Heart failure	124 (10.33)
Atrial fibrillation	115 (9.58)
Cancer	82 (6.83)
Chronic kidney disease	73 (6.08)
Chronic obstructive pulmonary disease	27 (2.25)
History of falls or fractures	25 (2.08)
Osteoporosis	19 (1.58)
Anxiety/depression	13 (1.08)
Parkinson disease	11 (0.92)
History of gastric or duodenal ulcers	9 (0.75)
Seizure	3 (0.25)

### 3.3 Polypharmacy

The median number of medications prescribed to the participants was 9 (IQR = 7–12), ranging from 1 to 27. Overall, 47.50% of the participants took 10 or more medications, and 44.08% took 5 to 9 medications (Table 1).

**Figure 1:**
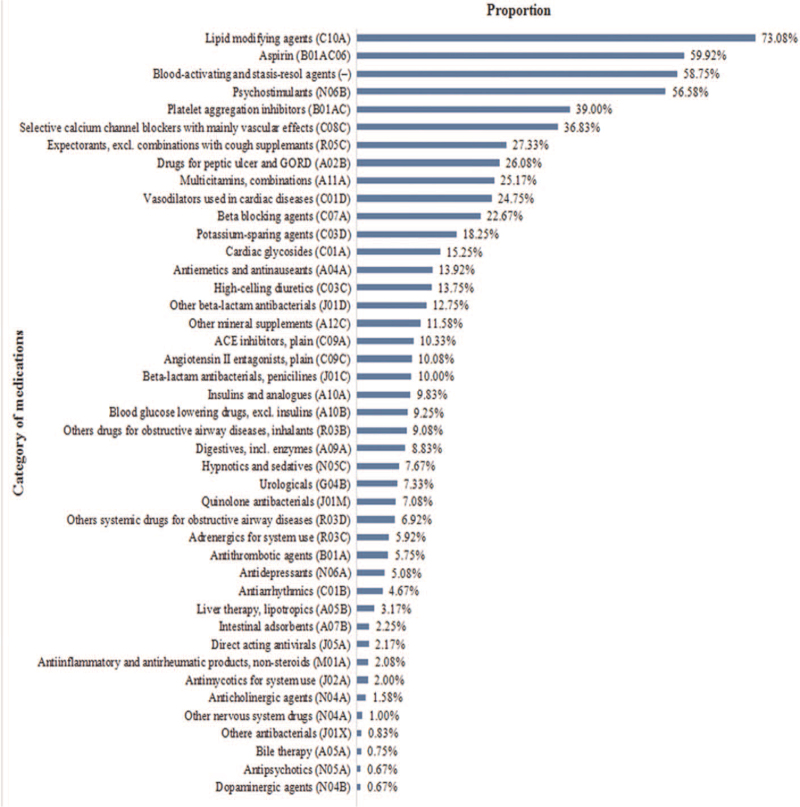
The proportion of study participants prescribed at least one medication, listed by the category of medication.

Lipid-lowering drugs were the most commonly used type of medication (n = 877, 73.08%) (Fig. 1). Aspirin (n = 719, 59.92%), as well as traditional Chinese patent drugs with the effect of promoting blood circulation and dispersing stasis (n = 705, 58.75%) were also commonly used. Some 18.16% (n = 128) of the patients were using 2 or more blood-activating and stasis-resoling agents, while 56.58% (n = 679) of the participants used psychostimulants, with 53.61% (n = 364) of these taking 2 or more psychostimulants, and 24.89% (n = 169) taking 3 or more.

### 3.4 PIMs use

The 2019 Beers criteria identified a total of 640 PIMs being used by 361 participants, accounting for 30.08% of the study population (Table 3). Among these participants, 196 (54.29%) took 2 or more PIMs. The number of PIMs taken by the 361 elderly participants ranged between 1 and 6. Among 640 PIMs, 1 PIM was noticed in 165 (13.75%) participants, 2 PIMs in 135 (11.25%), 3 in 46 (3.83%), 4 in 10 (0.83%), 5 in 3 (0.25%), and 6 PIMs in 2 (0.17%) participant. Roughly 30.00% of all male and female patients encountered PIMs, with participants in over 85 age group being subjected to the highest incidence of PIMs (42.67%) (Table 1).

**Table 3:** The prevalence of PIMs identified using the 2019 Beers criteria.

2019 Beers Criteria PIMs (n = 640)			
PIMs in older adults		n = 186	%
N05CF01	Zopiclone	78	41.94
C01BD01	Amiodarone	40	21.51
R06AD02	Promethazine	15	8.06
C01AA05	Digoxin	12	6.45
M01AB05	Diflunisal	8	4.30
N05CF04	Eszopiclone	8	4.30
N05AH03	Olanzapine	7	3.76
N05CD04	Estazolam	5	2.69
C08CA05	Nifedipine, immediate	3	1.61
N03AA02	Phenobarbital	2	1.08
N04AA01	Trihexyphenidyl	2	1.08
N05BA01	Diazepam	2	1.08
A03FA01	Metoclopramide	2	1.08
N05AA01	Chlorpromazine	1	0.54
N05CF02	Zolpidem	1	0.54

Drug-disease and drug-syndrome PIMS		n = 16	%
C08DB01	Diltiazem	8	50.00
M01AH01	Celecoxib	2	12.50
B01A009	Cilostazol	2	12.50
B01AC06	Aspirin	2	12.50
N02AX02	Tramadol	1	6.25
R06AD02	Promethazine	1	6.25

PIMs to be used with caution		n = 420	%
C03DA01	Spironolactone	217	51.67
C03CA01	Furosemide	165	39.29
B01AF01	Rivaroxaban	11	2.62
N06AB04	Citalopram	7	1.67
N02AX02	Tramadol	6	1.43
N06AB06	Sertraline	6	1.43
N06AX11	Mirtazapine	4	0.95
B01AC06	Aspirin	3	0.71
C03AA03	Hydrochlorothiazide	1	0.24

Drug–drug interactions		n = 8	%
N06AB06	Sertraline–olanzapine–eszopiclone	2	25.00
B01AA03	Theophyline–aspirin	1	12.50
B01AA03	Theophyline–amiodarone	1	12.50
N06AX11	Mirtazapine–estazolam–zopiclone	1	12.50
H02AB02	Dexamethasone–aspirin	1	12.50
N06AB04	Citalopram–zolpidem–zopiclone	1	12.50
N06AB04	Citalopram–estazolam–eszopiclone	1	12.50

PIMs based on kidney function		n = 10	%
C03DA01	Spironolactone	8	80.00
B01AF01	Rivaroxaban	1	10.00
B01AB05	Enoxaparin	1	10.00

Applying the 2019 Beers criteria, we found that the most frequently encountered PIMs category was “PIMs to be used with caution” (65.63%), followed by “PIMs in older adults” (29.06%), “drug-disease and drug-syndrome PIMs” (2.50%), “PIMs based on kidney function” (1.56%) and “drug–drug interactions” (1.25%, Table 3).

Details of the PIMs identified in this cross-sectional study are presented in Table 3. The most commonly identified PIM use in older adults was zopiclone, which may cause delirium, falls, and fractures with minimal improvement in sleep latency and duration. The most common drug-disease and drug-syndrome PIMs was diltiazem administrated for patients with heat failure, because diltiazem has the potential to promote fluid retention and exacerbate the disease. The most commonly encountered PIMs to be used with caution were spironolactone and furosemide, which may cause or exacerbate syndrome of inappropriate antidiuretic hormone secretion or hyponatremia. The majority of drug–drug interactions were antidepressants combined with 2 other drugs acting on the central nervous system, which increased falls. The most commonly encountered PIMs based on kidney function were spironolactone.

Twenty ADRs occurred in total participants. Of these, 18 ADRs due to PIMs were hyponatremia caused by diuretics (furosemide, spironolactone) (17) and selective serotonin reuptake inhibitors (sertraline) (1). The other 2 ADRs were gastrointestinal bleeding due to aspirin (1) and ticagrelor (1).

### 3.5 Factors associated with PIMs use

Compared to the non-PIM group, the PIM group demonstrated significant differences regarding age (*P* < .001), length of hospital stay (*P* = .001), the number of diagnosed disease (*P* < .001), and prescribed medication (*P* < .001) (Table 1). Multiple logistic regression analysis detected that hyperpolypharmacy was significantly associated with PIMs (Table 4). Compared with the prescription of 1 to 4 medications, the risk of PIM use for prescriptions of 10 or more medications soared to 12.44 (95% confidence interval [CI]: 5.69–27.19). In addition, for participants of 75 to 84 years old or those over 85 years of age, the risk of PIM use was increased to 1.69 or 2.23 fold, respectively, compared to the 65 to 74 age group. However, the longer length of hospital stay of ≥11 days group showed a lower risk compared to those with 1 to 5 days group (OR: 0.59, 95% CI: 0.38–0.93). No significant association was found between gender, number of diagnosed diseases, and PIM use in this study.

**Table 4:** The association between various factors and PIM use.

Variables	OR	95% CI	*P* value
Gender			
Male	1 (ref)	–	–
Female	0.886	0.67–1.17	.40
Age			
65–74	1 (ref)	–	–
75–84	1.69	1.25–2.27	.001
≥85	2.23	1.46–3.43	<.001
Length of hospital stay			
1–5	1 (ref)	–	–
6–10	0.79	0.52–1.18	.25
≥11	0.59	0.38–0.93	.02
No. diagnosed disease			
1–5	1 (ref)	–	–
6–10	1.34	0.97–1.85	.07
≥11	1.29	0.83–2.02	.25
No. prescribed medication			
1–4	1 (ref)	–	–
5–9	2.14	0.98–4.66	.06
≥10	12.44	5.69–27.19	<.001

### 3.6 Risk of PIM use across the number of prescribed medications

**Figure 2:**
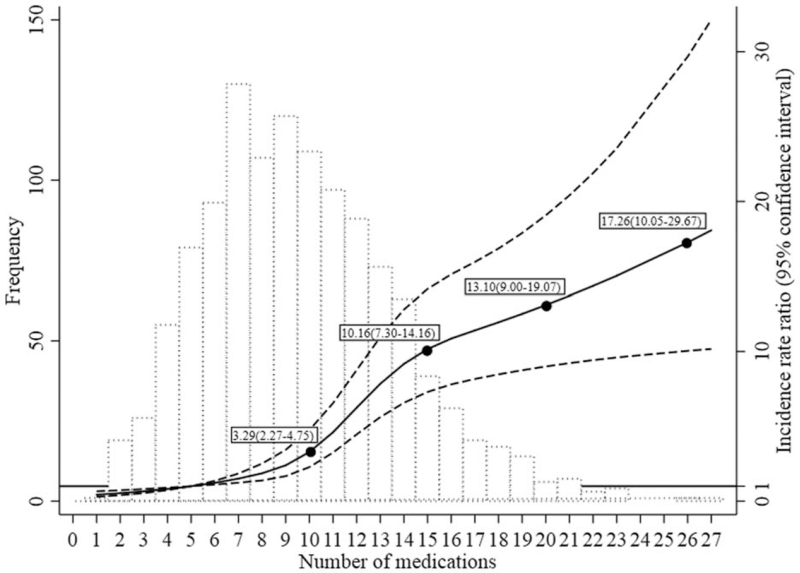
The association between the risk of PIM use and the number of prescribed medications. Histogram depicts the distribution of the number of prescribed medications in the study (left *y* axis). The solid black line (black dashed lines are 95% confidence intervals) is the adjusted incidence rate ratio, representing the average participant's risk of PIMs use across the number of prescribed medications, relative to the reference (5 medications). Model was adjusted for age, gender, length of hospital stay, and the number of diagnosed diseases. PIM = potentially inappropriate medication.

Just as the logistic regression analysis had determined, polypharmacy was the strongest predictor of PIM use by the zero-inflated negative binomial regression analysis. There was a nonlinear relationship between the number of prescribed medications and the risk of PIM use (Fig. 2). Compared to taking 5 medications, the use of 10 medications was associated with a 2.29 fold (95% CI = 1.27–3.75) higher risk of PIM use. Continuing the same trend, the use of 15 or 26 medications was associated with a 10.16 fold (95% CI = 7.30–14.16) or 13.10 fold (95% CI = 9.00–19.07) risk of PIM use, respectively, suggesting that the incidence of PIM use rises with the number of medications prescribed.

## 4 Discussion

In this retrospective, single-center, cross-sectional study of older adults, approximately half of the participants took 10 or more medications, with 30.33% of the study population being exposed to PIMs. Our findings suggested that hyperpolypharmacy and old age are the independent risk factors of PIM use. Minimizing unnecessary medication prescription may be a feasible approach to reducing PIM use in elderly patients.

The high prevalence of polypharmacy reported here is consistent with prior studies.^[8,30]^ In developed countries, about 20% of the elderly population and up to 70% of hospitalized older adults routinely take 5 or more medications.^[31]^ In 1 study conducted in China, 37.9% of elderly inpatients were regularly prescribed 10 or more medications.^[27]^ Similarly, 47.50% of the participants were reported as taking 10 or more prescribed medications during hospitalization in our study.

Widespread prevalence of PIM use has been previously documented within the Chinese elderly inpatients community. The present study investigated the prevalence of PIMs according to the latest 2019 Beers criteria, one of the most common explicit tools. The prevalence of PIMs assessed by the Beers criteria were reported higher than other criteria.^[25,32,33]^ A recent cross-sectional study conducted in western China used the 2012 Beers criteria to demonstrate that 43.4% of inpatients aged 65 to 79 years and 58.2% aged 80 years or older consumed one or more PIMs.^[24]^ More than half of the older inpatients were observed to have at least 1 PIM in 2 past studies.^[27,34]^ Our study highlighted that 30.33% of inpatients aged 65 or older were exposed to PIMs, which was lower than the percentage reported by aforementioned studies. This may be related to the low usage rate of drugs listed in 2019 Beers criteria, as well as the discrepancies in the geographical location, the disease characteristics of patients, the prescription behavior of physicians, and the specific hospital drugs list.

The most frequently encountered PIMs should be used with caution in this study were diuretics (383 PIM use, corresponding to 59.84% of all PIM use), and it was comparable to 1 study conducted in China.^[27]^ Spironolactone and furosemide are typically used in patients with fluid retention.^[35]^ PIM use involving diuretics was also identified in older patients with cardiovascular disease in 1 US study.^[10]^ Besides diuresis, a condition associated with excessive production of urine, long-term therapy with diuretics may cause or exacerbate syndrome of inappropriate antidiuretic hormone secretion or hyponatremia. According to the 2019 Beers criteria, the use of diuretics is to be used with caution, while sodium levels should be closely monitored when starting or changing the dosages of these drugs in elderly. We also found that the participants in our study were exposed to high levels of PIMs belonging to the class of non-benzodiazepine hypnotics (87 PIMs use, corresponding to 13.59% of all PIM use). In contrast, Komagamine^[36]^ and Mo et al^[24]^ showed that benzodiazepines were the most common PIM use among the elderly inpatients in Japan and China, respectively. Given the harmful side-effects and minimal improvement in sleep latency and duration exhibited by non-benzodiazepine hypnotics, this type of medication should be used with caution, especially in elderly patients.

It is common for geriatric patients to demonstrate multimorbidity, requiring multiple drugs for the treatment of each individual disease, in order to harness the best clinical benefits. In addition, many elderly individuals in China are convinced that taking medicine is the best choice.^[24]^ This study established that PIM use is significantly associated with polypharmacy and older age. Furthermore, polypharmacy seems to be the most evident risk factor of PIMs, which is in accordance with the findings of previous study.^[27]^ However, it is hard to determine the extent of polypharmacy based on the number of medications prescribed, owing to the lack of standards. Even so, this study has revealed the nonlinear relationship between the number of medications taken by a patient and their risk of PIM exposure.

More recently, deprescribing has been proven to be effective in reducing polypharmacy and PIMs in older patients.^[31,37–40]^ Therefore, systematic strategies should be implemented to minimize polypharmacy, by stopping the inappropriate prescribing of unnecessary medications. It is essential to encourage clinicians to consider the risks and benefits when prescribing medications to older patients, especially those with polypharmacy. The development of elderly patient-centered educational programs on drug use, as well as collaborative prescriber–pharmacist reviews of drugs, should be promoted to mitigate the use of PIMs.

Several limitations can be noted in this study. Firstly, the 2019 Beers criteria were applied to identify PIMs in this study due to the lack of local standards. Beers criteria refer to a relative assessment of PIM use risk in the elderly but do not account for the PIMs unique to traditional Chinese patent medicine. Secondly, it was a retrospective cross-sectional study without follow-up, and the analysis was based on EMR-recorded data. Finally, this is a single-center, small sample size study that may represent, but cannot be generalized to the whole of China.

## 5 Conclusion

A high prevalence of polypharmacy and PIM use in elderly patients was observed. Polypharmacy and older age were identified as independent factors associated with PIMs. Moreover, there was a significant association between the extent of polypharmacy and the risk of PIM use. Systematic strategies should therefore be adopted to minimize polypharmacy and mitigate PIM use.

## Acknowledgments

The authors gratefully thank Lixin Tao for the statistical guidance at School of Public Health Capital Medical University.

## Author contributions

**Conceptualization:** Lina Tao, Yanqing Song.

**Data curation:** Xiaoyu Qu.

**Methodology:** Huan Gao, Yanqing Song.

**Software:** Xiaoyu Qu.

**Supervision:** Jinghui Zhai.

**Validation:** Yueming Zhang.

**Writing – original draft:** Lina Tao, Yanqing Song.

**Writing – review & editing:** Yueming Zhang.
